# Successful rescue of renal transplantation with cardiac arrest after electrical storm: A case report

**DOI:** 10.1097/MD.0000000000032030

**Published:** 2022-11-25

**Authors:** Hao Li, Zhiping Xia, Ling Li, Zhongshan Lu, Futian Du, Qifa Ye, Guizhu Peng

**Affiliations:** a Zhongnan Hospital of Wuhan University, Institute of Hepatobiliary Diseases of Wuhan University, Transplant Center of Wuhan University, Hubei Key Laboratory of Medical Technology on Transplantation, Wuhan, Hubei, China; b Weifang People’s Hospital, Hepatobiliary and Pancreatic Medicine Center, Weifang Shandong, China.

**Keywords:** cardiac arrest, cardiopulmonary resuscitation, electrical storm, renal transplantation

## Abstract

Most patients with end-stage chronic kidney disease are associated with complications such as renal hypertension, renal anemia, hyperkalemia, water-sodium retention, and disorders of acid-base balance after long-term renal replacement therapy, which can lead to increased cardiac burden, some degree of myocardial damage, and finally progress to arrhythmia and heart failure. These are the main reasons why patients with chronic kidney disease are prone to cardiovascular events after renal transplantation.

**Patient concerns:**

We report a case of sudden onset of ventricular fibrillation on the postoperative second day, with repeated electrical storm accompanied by cardiac arrest during resuscitation, a very long cardiopulmonary resuscitation (CPR) process of 5 hours and 14 minutes, and >20 cycles of cardiac defibrillation.

**Diagnoses:**

According to the patient history and resuscitation process, a diagnosis of ES with cardiac arrest after renal transplantation was formulated.

**Intervention:**

According to the American Heart Association guidelines for CPR and cardiovascular emergencies, resuscitation measures such as CPR, tracheal intubation, electric defibrillation, symptomatic medication, etc. were performed on the patient.

**Outcomes:**

Finally, the patient was successfully resuscitated, after which the patient had stable respiratory circulation and no neurological complications. To our knowledge, this is the only reported case in which a patient survived with good neurologic outcomes after a resuscitation that lasted as long as 5 hours and 14 minutes.

**Lessons:**

This case of adequate resuscitation can provide experience and a basis for CPR of patients with in-hospital complications of cardiovascular events for a long time.

## 1. Introduction

Electrical storm (ES) is an acute critical syndrome of >2 to 3 ventricular tachycardia or ventricular fibrillation within 24 hours, causing hemodynamic disturbances and requiring immediate electrical cardioversion or defibrillation,^[1]^ usually in patients with myocardial infarction or non-ischemic cardiomyopathy; its high mortality rate is one of the most challenging clinical manifestations in the cardiac field.^[2]^ The high mortality rate is one of the most challenging clinical presentations in the cardiac neighborhood.^[3]^ Most patients with end-stage chronic kidney disease have some degree of myocardial damage due to increased long-term cardiac load,^[4]^ this is the main reason why patients with end-stage chronic kidney disease are prone to cardiovascular events after renal transplantation.^[5]^ Renal transplantation is the most effective treatment for curing chronic end-stage renal disease and eliminating ongoing cardiovascular damage in patients^[6]^; however, cardiovascular events account for 20% of deaths after renal transplantation.^[7]^ The rare occurrence of ES with an accompanying heartbeat after renal transplantation, which is dangerous, difficult to resuscitate, and often leaves sequelae, is a significant prognostic challenge in renal transplant patients. We present a case of how recent research findings in resuscitative medicine improved 1 patient’s chances of survival.

## 2. Case report

A 44-year-old man weighing 120 kg was admitted to our hospital on September 18, 2018. This was because he had discovered that his creatinine level had been elevated for 5 years and was on regular hemodialysis treatment for 18 months (3 times/week). The patient had a 10-year history of diabetes mellitus with hypertension and no history of angina attacks. The patient was diagnosed with end-stage chronic kidney disease and was undergoing hemodialysis after relevant investigations and evaluations. On September 20, he underwent hemodialysis treatment. On September 23, the patient received a kidney transplant from a deceased donor. The kidneys were obtained by a Chinese organ procurement organization physician and allocated by the Chinese Human Organ Transplant Response System. Several pre-operative tests were performed.

**Figure 1.**
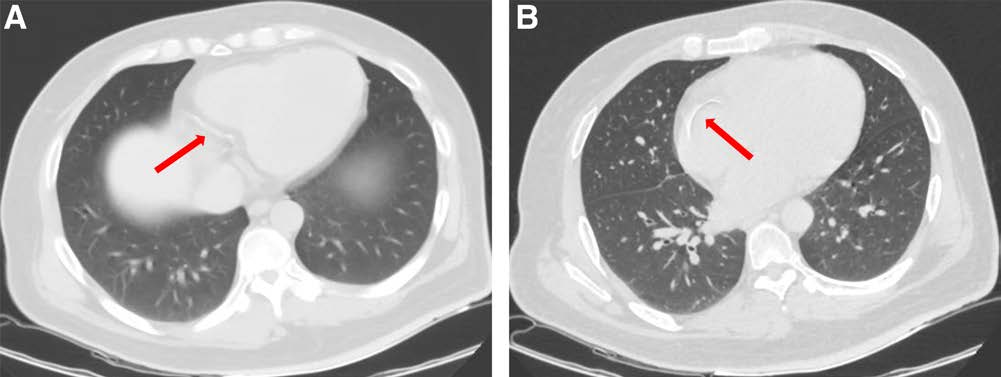
(A and B) Preoperative chest computed tomography (CT) scan, The red arrow points to coronary arteriosclerosis.

**Figure 2.**
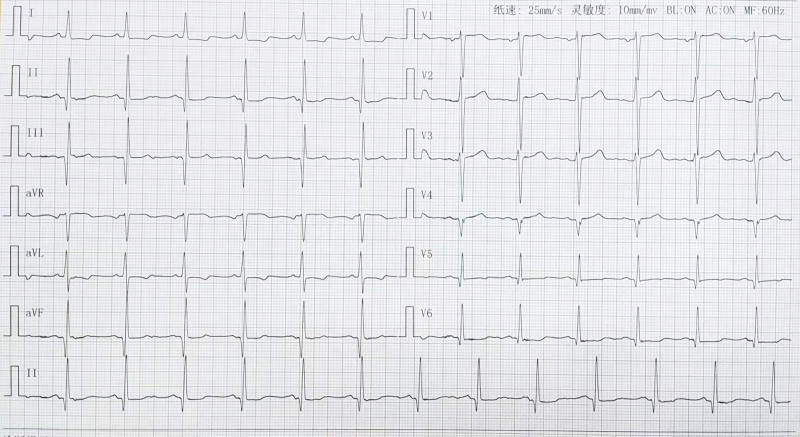
Preoperative electrocardiogram (ECG).

The peripheral white blood cell count was 7.31 × 10^9^/L, red blood cell (RBC) count was 3.68 × 10^12^/L, hemoglobin was 123.7 g/L, platelets 130 × 10^9^/L, lymphocyte count 1.36 × 10^9^/L, and serum creatinine level 836 μmol/L.
Blood type A (Rh-positive), 5 human leukocyte antigen (HLA) mismatch sites, negative panel reactive antibodies, negative lymphocytotoxicity test.
Pulmonary computed tomography showed aortic and coronary artery atherosclerosis (Fig. 1A and B).
Electrocardiography (ECG) showed ST-T changes in leads v1 and v3 and old inferior and anterior lateral wall myocardial infarction (Fig. 2).

A total of 5 U of concentrated RBCs, 350 mL of plasma, and 7500 mL of fluid were transfused intraoperatively and upon return to the transplant intensive care unit post-operative. Immunosuppressive therapy consisted of oral tacrolimus (2 mg q12h), mycophenolate mofetil (250 mg q12h), and intravenous methylprednisolone (500 mg qd). Cefoperazone sodium and sulbactam sodium (2 g, q12h) were used to prevent infection. The urine output on post-operative first day was 1525 mL. RBC 1.98* 10^12^/L, hemoglobin 67.3 g/L.

The patient experienced a sudden loss of consciousness on 5:24 September 25, 2018 (25 hours post-operative). ECG monitoring showed ventricular fibrillation and a sharp drop in oxygen saturation, followed by cardiac compressions, mask oxygenation, defibrillation by electric shock, administration of epinephrine 1 mg by static push, and urgent check of the patient’s arterial blood gases and electrolytes. The patient’s percutaneous oxygen saturation (SpO2) continued to decrease to 50%. Emergency tracheal intubation was changed to ventilator-assisted breathing at 5:35, ventilation mode was volume support ventilation, tidal volume was 550 mL, frequency was 12/minute. Urgent check of the patient’s blood gas and electrolyte results: pH = 7.273, blood potassium 5.8 mmol/L. Insulin 8U + 50% glucose injection 60 mL and 5% sodium bicarbonate 250 mL were administered. The initial resuscitation lasted nearly 40 minutes, cardiac compressions could bring blood pressure to the normal range, and blood oxygen saturation could be maintained above 85% under ventilator-assisted breathing. During resuscitation, norepinephrine and atropine were pumped intravenously to maintain blood pressure, while intravenous blood and fluid were transfused. When ventricular fibrillation occurred, lidocaine 5 mg and amiodarone 300 mg were administered intravenously. Until the first successful cardiopulmonary resuscitation (CPR) at 08:40, the ECG monitor showed a stable waveform in the patient, and consultation with a cardiovascular physician was requested. The consultation advice was as follows: perform airway management, adjust the respiratory parameters to make the rate of breathing to 10/minute, maintain SpO2 at 92% to 98%, and partial pressure of carbon dioxide at 35 to 45 mm Hg; perform hemodynamic management to maintain blood pressure at 90/65 mm Hg; and perform target temperature management target to maintain 33 to 36°C. Ventricular fibrillation reappeared at 09:15, the patient was again defibrillated by electric shock, and cardiac compressions continued. At this stage of resuscitation through high-quality CPR, blood pressure and oxygen saturation can be maintained in the normal range, the last defibrillation time at 10:38, after the resumption of voluntary respiration and pulse, from extracardiac compressions, blood pressure, and oxygen saturation can still be maintained.

**Figure 3.**
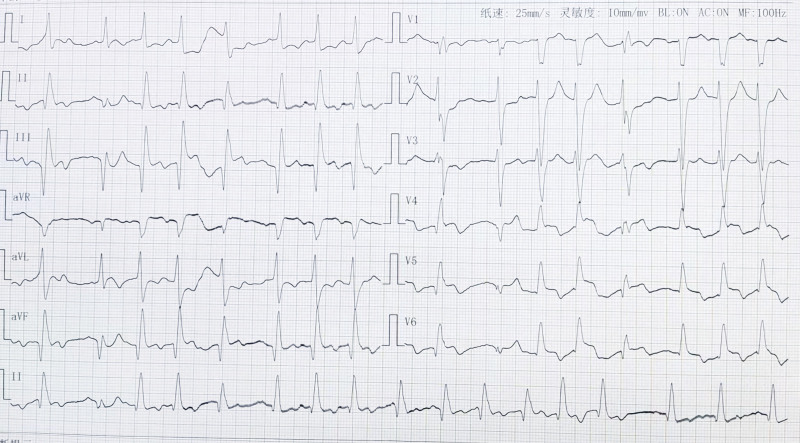
The electrocardiogram (ECG) of the day after successful cardiopulmonary resuscitation (CPR).

Resuscitation lasted 5 hours and 14 minutes from the first cardiac arrest at 5:24 hours to the resumption of the last defibrillated heart. During this process, cardiac compressions were not stopped, epinephrine was started at 1 mg and then repeatedly pushed according to the patient’s needs, and >20 defibrillations were recorded. At 15:00 on the same day, the patient regained consciousness and was able to respond to calls; his blood pressure was stable, and he was able to reach a blood oxygen saturation of >90% after trying to stop the ventilator and switch to spontaneous breathing, and his respiratory circulation was stable after removal of tracheal intubation. The patient continued to receive vasoactive drugs and nasal catheter oxygen to maintain respiratory circulation stability and to reduce neurological damage. The results of the repeat electrocardiogram showed that sinus rhythm and inferior and anterior lateral wall myocardial infarction could not be excluded; some leads had ST-T interval changes (Fig. 3). One week after the treatment, the patient was transferred from the transplant intensive care unit to the general transplant ward.

## 3. Discussion

In this case, the increased cardiac load due to long-term renal failure and dialysis treatment and renal hypertension, which caused some degree of myocardial damage,^[5]^ and the possible cardiovascular vascular pathology in patients with combined diabetes mellitus are the main reasons why patients with chronic kidney disease are prone to cardiovascular events after renal transplantation. Despite the presence of coronary atherosclerosis and old infarction on preoperative chest computed tomography and electrocardiogram, emergency matching of a suitable kidney source by Chinese Human Organ Transplant Response System and the need for emergency surgery due to the limitation of organ preservation time are potential risks for post-operative cardiovascular events. In addition, excessive intraoperative bleeding aggravates the risk of post-operative cardiovascular accidents, and improper perioperative control of fluid volume and disturbance of electrolyte and acid-base balance are direct factors that exacerbate ES with cardiac arrest in the post-operative period.^[8]^ Therefore, close attention should be paid to the electrolyte profile and volume control in patients after renal transplantation, because fluid imbalance or electrolyte disturbance can induce cardiac arrhythmia or even cardiac arrest. HLA mismatch or poor donor kidney quality is closely related to postoperative cardiovascular events.^[9]^ However, the excellent quality of the transplanted kidney and the good recovery of urine output after surgery in this case, multiple point mismatches in the preoperative HLA mismatch, were not obvious causes of the patient’s cardiac arrest. The main reason for cardiac arrest may still be related to multiple factors, such as cardiovascular vascular disease, chronic impairment of cardiac function, excessive surgical blood loss, electrolyte and acid-base balance disorders, and improper fluid rehydration control. Physicians were required to perform relevant preoperative tests to avoid cardiovascular events after renal transplantation, including echocardiography, ECG, and coronary artery CTA, and to perform an adequate preoperative evaluation together with anesthesiologists.

In the United States, the percentage of out-of-hospital cardiac arrest patients who achieve good functional status for survival is approximately 8.2%, while the survival rate at discharge for in-hospital cardiac arrest is approximately 25.8%, with 82% of these patients having good neurological status at discharge.^[10–13]^ The patient was undergoing post-transplant symptomatic treatment in the transplant intensive care unit; therefore, when the patient developed ventricular fibrillation, bedside nurses were able to detect and report it to the on-duty physician in a timely manner. With ECG monitoring, finger pulse oxygen monitoring, and continuous arterial blood pressure monitoring, the timing of initiating CPR was more precise for the medical staff. With intravenous access established in the patient’s body, the nursing team can quickly administer symptomatic treatment according to the physician’s orders while the physician resuscitates the patient. When the oxygen saturation of the patient could not be elevated by the initial mask administration, tracheal intubation was promptly performed to establish an advanced airway, which could provide a constant tidal volume and oxygen concentration for the patient after connecting to the ventilator. A manual electric defibrillator was used during the pre-resuscitation period to induce electric shock defibrillation when ventricular fibrillation occurred. Since the patient had frequent ventricular fibrillation, it was switched to a Zoll automatic external defibrillator that could monitor specific arrhythmias in real time and provide accurate and timely defibrillation. Moreover, it was able to correct ineffective external cardiac compressions during CPR.^[14]^ The use of this defibrillator was guaranteed to provide high-quality defibrillation and resuscitation despite prolonged resuscitation, and was a major reason for successful resuscitation. After 3 failed defibrillation treatments, epinephrine was administered immediately. Studies have shown that in patients with cardiac arrest, early administration of epinephrine improves survival, with a 4% improvement in survival for each 1 minute advance in epinephrine administration.^[15]^ We also administered high doses of intravenous amiodarone and lidocaine as antiarrhythmic therapy. Amiodarone is widely used for recurrent arrhythmias and has been reported to be effective as a stand-alone drug intravenously (1 g/day) to terminate ventricular fibrillation cycles within 24 hours, and its effectiveness in ES therapy has been demonstrated.^[16]^ In addition to these drugs, sodium bicarbonate, high glucose + insulin, norepinephrine, and atropine are also administered for related symptomatic management, as their application maintains blood pressure, corrects electrolyte disturbances and acid-base imbalance, and minimizes damage to all organs of the body, especially the nervous system. Some studies have also pointed out that prolonged electrical resuscitation may damage cardiac myocytes and require prompt other evaluations, such as catheter ablation, but the current data support early intervention.^[17]^ In contrast, extracorporeal pulmonary oxygenation support is a good option for stabilizing patients with severe ES in electrical storms accompanied by cardiac arrest, providing valuable time for effective treatment.^[18]^

The successful resuscitation in this case was attributed to early detection, timely resuscitation, and close cooperation of medical and nursing staff. The intensive care unit provided good resuscitation conditions, target temperature management, establishment of advanced airway, prolonged chest compressions, timely and effective electric defibrillation, and symptomatic support treatment with drugs that were crucial to maintain the stability of respiratory circulation, which significantly improved the rescue rate of cardiac arrest and reduced brain injury in the patient. The duration of a single arrest with complete absence of cardiac activity during resuscitation in this case far exceeded the time limit of 20 minutes considered by the guidelines to abandon resuscitation,^[19]^ and a total resuscitation time of 5 hours and 14 minutes with successful resuscitation has not yet been reported in the literature, thus providing clinicians with some experience and basis for prolonged resuscitation of in-hospital patients with cardiovascular events.

HL and ZX contributed equally to this work.

## Acknowledgments

We thank the doctors, nurses, and clinical staff who provided care for the patients.

## Author contributions

Xia Zhiping (MD) and Peng Guizhu (MD) rescued the patient, and Li Hao drafted this manuscript. All authors contributed to the review and final approval of this manuscript.

**Formal analysis:** Hao Li, Zhiping Xia, Zhongshan Lu, Guizhu Peng.

**Funding acquisition:** Zhiping Xia, Guizhu Peng.

**Resources:** Guizhu Peng.

**Software:** Guizhu Peng.

**Writing – original draft:** Hao Li, Guizhu Peng.

**Writing – review & editing:** Hao Li, Zhiping Xia, Ling Li, Zhongshan Lu, Futian Du, Qifa Ye, Guizhu Peng.
